# Application of Machine Learning Technique to Distinguish Parkinson’s Disease Dementia and Alzheimer’s Dementia: Predictive Power of Parkinson’s Disease-Related Non-Motor Symptoms and Neuropsychological Profile

**DOI:** 10.3390/jpm10020031

**Published:** 2020-04-28

**Authors:** Haewon Byeon

**Affiliations:** Department of Speech Language Pathology, School of Public Health, Honam University, 417, Eodeung-daero, Gwangsan-gu, Gwangju 62399, Korea; bhwpuma@naver.com; Tel.: +82-10-7404-6969

**Keywords:** Alzheimer’s dementia, Parkinson’s disease dementia, cognitive function, random forest, MoCA, neuropsychological profile

## Abstract

In order to develop a predictive model that can distinguish Parkinson’s disease dementia (PDD) from other dementia types, such as Alzheimer’s dementia (AD), it is necessary to evaluate and identify the predictive accuracy of the cognitive profile while considering the non-motor symptoms, such as depression and rapid eye movement (REM) sleep behavior disorders. This study compared Parkinson’s disease (PD)’s non-motor symptoms and the diagnostic predictive power of cognitive profiles that distinguish AD and PD using machine learning. This study analyzed 118 patients with AD and 110 patients with PDD, and all subjects were 60 years or older. In order to develop the PDD prediction model, the dataset was divided into training data (70%) and test data (30%). The prediction accuracy of the model was calculated by the recognition rate. The results of this study show that Parkinson-related non-motor symptoms, such as REM sleep behavior disorders, and cognitive screening tests, such as Korean version of Montreal Cognitive Assessment, were highly accurate factors for predicting PDD. It is required to develop customized screening tests that can detect PDD in the early stage based on these results. Furthermore, it is believed that including biomarkers such as brain images or cerebrospinal fluid as input variables will be more useful for developing PDD prediction models in the future.

## 1. Introduction

Parkinson’s disease (PD) is a representative geriatric neurodegenerative disease that is characterized by motor symptoms such as rest tremor, rigidity, bradykinesia, and postural instability [1]. It is known that it is due to dopamine deficiency (≥50%) in the substania nigra and the pars compacta [1]. The incidence rate of PD was reported to be 12,500 per 100,000 people [2]. In the United States, the incidence rate of PD steadily increased for 30 years [3]. Since PD has traditionally been considered as dyskinesia caused by dopamine deficiency, the cognitive impairments of PD have received relatively less attention. However, PD may cause non-motor symptoms such as cognitive dysfunction, depression, and rapid eye movement (REM) sleep behavior disorders in the course of development [4] and these symptoms are highly likely to adversely affect the quality of patients’ lives. [5]. As a result, more recent studies examined the non-motor symptoms of PD.

The key of non-motor symptoms related to PD is the decline of cognitive function, which affects the quality of a patient’s life. Previous studies have shown that 80% of patients with PD had cognitive impairment [6]. For example, Hely et al. (2008) [7] conducted an epidemiological study that tracked PD patients for over 20 years. They found that 84% of them developed cognitive impairment and 50% of them were developed to Parkinson’s disease dementia (PDD). It has also been reported that people with PD have a six times higher risk of developing dementia than healthy elderly [8]. PDD is the second most common degenerative brain disease following Alzheimer’s disease (AD) [9,10]. PDD increases the care burden as well as mortality or morbidity [11]. Therefore, it is clinically important to detect PDD as soon as possible. 

Since incipient dementia does not have any major defects in cognitive function for maintaining daily life, it is difficult to discriminate it from age-associated cognitive decline or cognitive frailty [12,13]. The biggest barrier to diagnose PDD early is that patients do not suspect PDD [14]. Patients tend not to voluntarily report initial symptoms other than the main symptoms that they are aware of [14]. Therefore, even if a slight cognitive decline is found in the patient, it is hard for medical personnel to distinguish it from cognitive decline in the natural aging process [14]. Furthermore, it is difficult to clearly identify the presence or absence of each symptom because various non-motor symptoms of PDD appear at the same time at a certain point [15]. Therefore, it is critical to use a neuropsychological screening test, which has high accuracy and reliability and can be used easily by medical personnel in a short time, to identify dementia early. To achieve this goal, it is necessary to explore test indicators with a high diagnostic predictive capacity for dementia.

Nevertheless, further studies are needed to identify and evaluate indices that can distinguish PDD from other types of dementia such as AD. Although previous studies [16,17,18] reported that the distinctive characteristics of PDD were the deterioration of executive function from the cognitive function test and problems associated with free recall in the memory test, Péran et al. (2003) [19] reported that PD without dementia could have decreased executive functions such as worsened verbal fluency. However, AD and other types of cognitive disorders also show problems associated with executive and memory functions in the early stage. Therefore, it is difficult to distinguish PDD from other dementia types simply based on executive functions. Although it is known that patients with AD mainly suffer from the damage of cortical profile (e.g., language ability and memory) and those with PDD have defects in the subcortical profile (e.g., visuospatial ability and executive function) [20], it is still possible that a lot of their cognitive impairments may overlap. Additionally, Song et al. (2008) [21] reported that 26% of PDD cases had cognitive impairments that were similar to AD and Mann (1990) [22] revealed that 50% of PD without dementia had amyloid accumulation, which is a well–known characteristic of AD. The risk of dementia increases when the patients of PD are older and the onset of PD is later [23]. If the atrophy of the hippocampus is confirmed on brain magnetic resonance imaging, it is possible that PD and AD occur at the same time. In this case, it is not clear whether PD precedes AD or not, so it is more difficult to differentiate between PDD and AD.

According to the guideline (2007) [24] of the movement disorder society task force, which established the diagnostic definition of PDD, it is recommended to conduct level II, which is composed of 42 different neuropsychiatric test tools testing various functions, including instrumental functions, neuropsychiatric functions, and executive functions for precise diagnosis of PDD. However, it is difficult to conduct all the test tools suggested by level II in a short time in the clinical condition. In addition, it has been reported that the impaired neuropsychological functions of PD are influenced by factors such as depression, the severity of speech disorders, and REM sleep behavior disorders in addition to the time of onset and age [25,26]. Therefore, in order to develop a predictive model that can distinguish PDD from other dementia types, such as AD, it is necessary to evaluate and identify the predictive accuracy of the cognitive profile while considering the depression and REM sleep behavior disorders of PD.

In recent years, numerous studies have utilized data mining techniques to predict high-risk groups of diseases in the medical field [27]. Traditionally, tree-based methods have been used as algorithms to develop disease prediction models [28]. However, the accuracy of a generated decision tree may vary greatly depending on an input variable and a decision tree has the possibility of overfitting. In order to overcome these limitations, recent studies mainly use random forest, which performs random sampling on the same data set to generate multiple decision trees and predict final target variables by combining them, to predict diseases [29,30]. As far as we are aware, there is no study analyzing the diagnosis predictive power of cognitive profiles that can distinguish between AD and PDD considering PD non-motor symptoms such as depression and REM sleep behavior disorders in addition to known cognitive functions. This study compared PD’s non-motor symptoms and the diagnostic predictive power of cognitive profiles that distinguish AD, cortical dementia, and PD, subcortical dementia using machine learning.

## 2. Methods and Materials

### 2.1. Data Source

This study was carried out using the Parkinson’s Dementia Clinical Epidemiology Data (PADEM-data) from the National Biobank of Korea, the Center for Disease Control and Prevention, the Republic of Korea (No.KBN-2019-005). This study was approved by the Research Ethics Review Board (No. KBN-2019-005), the National Biobank of Korea, and it was also approved by the Korea Centers for Disease Control and Prevention to use the data (No. KBN-2019-1327). The National Biobank of Korea was founded in 2008 upon the approval of the Ministry of Health and Welfare and governed by the Korea Centers for Disease Control and Prevention as the necessity of managing medical data systematically at the national level has emerged. The primary goal of the National Biobank of Korea is to advance biomedical research and public health. Please refer to Lee et al. [31] for further details of the National Biobank of Korea, including quality control programs. 

The PADEM-data were collected under the management of the Korea Centers for Disease Control and Prevention at 14 tertiary care organizations (university hospitals) from Jan to Dec, 2015. Health surveys were performed using computer-assisted personal interviews. The data were composed of sociodemographic factors, health behaviors, disease history, motor characteristics related to PD, REM sleep behavior disorders, and neuropsychological test results [32]. Patients with idiopathic PD were diagnosed by the neurologists according to the diagnostic criteria of the United Kingdom Parkinson’s disease Society Brain Bank [33]. In this study, PDD was defined as patients who met the diagnostic criteria of probable PDD suggested by the Movement Disorder Society Task Force [24] among them. This study excluded patients who had other causative diseases, such as hydrocephalus and vascular Parkinsonism besides PD from MRI. AD was defined by using the criteria of the National Institute of Neurologic and Communicative Disorders and Stroke and Alzheimer’s Disease and Related Disorders Association (NINCDS-ADRDA). This study analyzed 118 patients with AD and 110 patients with PDD and all subjects were 60 years or older.

### 2.2. Measurement

The outcome variables were defined as PDD classified by medical diagnosis. The input variables included rapid eye movement REM sleep behavior disorders, and neuropsychological characteristics (the Korean version of Mini Mental State Examination (K-MMSE) [34], Korean version of Montreal Cognitive Assessment (K-MoCA) [35], the Geriatric Depression Scale (GDS) [36], Global Clinical Dementia Rating (CDR) score [37], Korean version of Instrumental Activities of Daily Living (K-IADL) [38]).

### 2.3. Development and Evaluation of Random Forest

In order to develop the PDD prediction model, the data were separated into training data (70%) and test data (30%). The random forest algorithm was used to develop the prediction model, and the performance of the developed prediction model was compared with that of multiple logistic regression and Classification and Regression Tree (CART). The accuracy of the prediction model was calculated by the recognition rate

**Figure 1:**
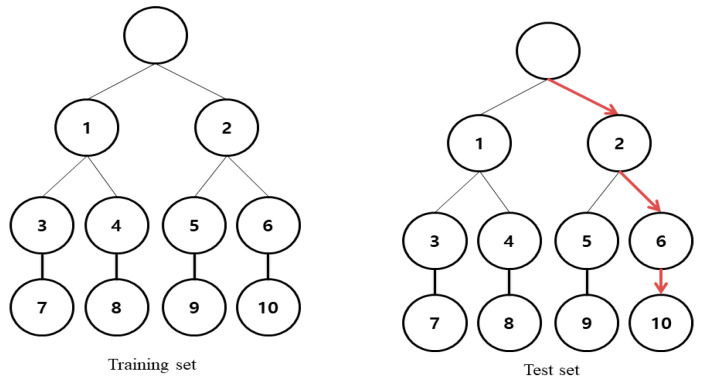
Examples of training and testing set.

**Figure 2:**
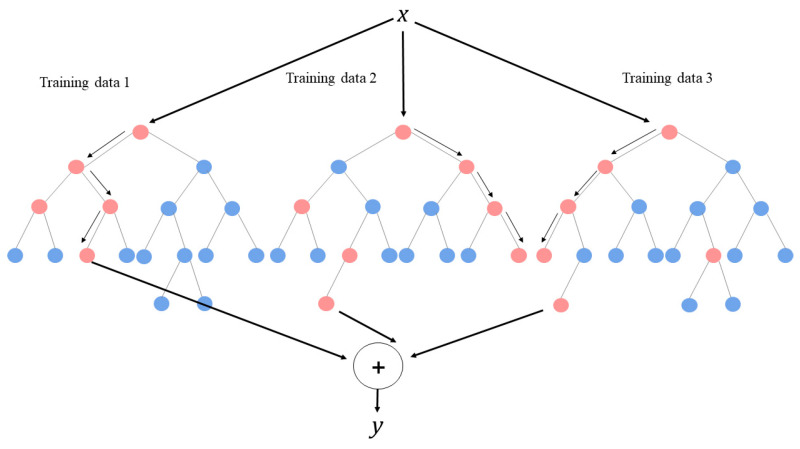
Classifier that combines many single decision trees.

Random forest is one of the ensemble classifiers. Random forest randomly learns multiple decision trees. It consists of a training step, generating many decision trees, and a test step, classifying or predicting predictors’ input vectors (Figure 1). The ensemble form of the training data can be expressed as Forest F = {f1, … , fn} (Figure 2). The distributions gained from the decision trees of each forest are averaged by T (the number of the decision trees) and the final classification is performed based on the results. The predictors of each sample were combined by using a mean value when a target variable is a continuous variable and using a majority vote when a target variable is a categorical variable. The function is presented in Equation (1).

(1) $$L\left(p\right)=\frac{1}{T}\;\sum_{t=1}^{T}P_{t}\left(b|I,\;p\right)$$

Random forest is similar to the bagging technique in the aspect of combining decision trees generated from numerous bootstrap samples using the majority vote principle to increase the stability, but they are different because the former uses a few explanatory variables that were randomly selected from each bootstrap sample. Random forest builds decision trees by randomly extracting explanatory variables from a bootstrap sample to control the correlation between combined models. However, pruning is minimized while constructing models. Random forest can be free from overfitting theoretically and is not affected by noise or outliers much [32]. Moreover, it can generate high accuracy results by reducing generalization errors. However, random forest has a higher probability that each tree will be more complex when an unimportant explanatory variable is selected [32]. Therefore, this study improved the accuracy of the model by considering the number of mtry, the number of candidate explanatory variables, in advance. This study changed mtry values (numbers), presenting the number of explanatory variables to be used in the decision tree constituting random forest, from 1 to 10, and selected value with the smallest error of out-of-bag.

This study predicted the main explanatory variables regarding the dependent variables of random forest using the importance of variables. The relative importance of variables means the degree of an explanatory variable’s effects on the classification accuracy of a model and it is calculated by using Gini impurity. If an explanatory variable improves the classification performance of a model considerably, the Gini impurity of this variable will decrease and the importance of this variable will increase. The function of the Gini impurity is presented in Equation (2).

(2) $$G\left(p_{1},\;p_{2},\;\cdots ,\;p_{J}\right)=\sum_{i=1}^{J}p_{i}\left(1-p_{i}\right)$$

This study first generated a random forest model and then compared the accuracies of models with the results obtained from multivariate logistic regression and decision trees. This study conducted statistical analyses using the ’RandomForest’ package of R version 3.6.2 (Foundation for Statistical Computing, Vienna, Austria).

## 3. Results

### 3.1. General Characteristics of Subjects

Among 228 subjects, 51.8% were patients with AD and 48.2% were patients with PDD. Males were 33.8% and females were 66.2%. The mean age was 74.6 years (range = 61−94 years; standard deviation = 6.5). The common education level of subjects was middle school graduate or below (73.2%). It was found that 2.2% of the subjects had a family history of PD and 4.4% had a family history of AD. Moreover, 4.2% had a history of head injury and 5.8% had carbon monoxide poisoning. Additionally, 21.9%, 46.3%, 8.3%, and 3.7% of the subjects had diabetes, high blood pressure, hyperlipidemia, and atrial fibrillation, respectively.

### 3.2. Characteristics of Subjects by Dementia Type

The general characteristics of the subjects, by the type of dementia, are presented in Table 1. The results of the chi-square test show that subjects were significantly different in terms of age and PD family history (*p* < 0.05). The characteristics of Parkinson’s non-motor symptoms and neuropsychological tests by dementia type are presented in Table 2. PDD had a significantly (*p* < 0.05) higher prevalence of REM sleep behavior disorders (76.1%) and depression (62.1%). The mean K-MoCA score of PDD was significantly (*p* < 0.05) higher than that of AD. However, AD had significantly (*p* < 0.05) higher global CDR score and sum of boxes in CDR than PDD.

### 3.3. Diagnostic Predictive Capability Using Random Forest for Distinguishing between AD and PDD

**Figure 3:**
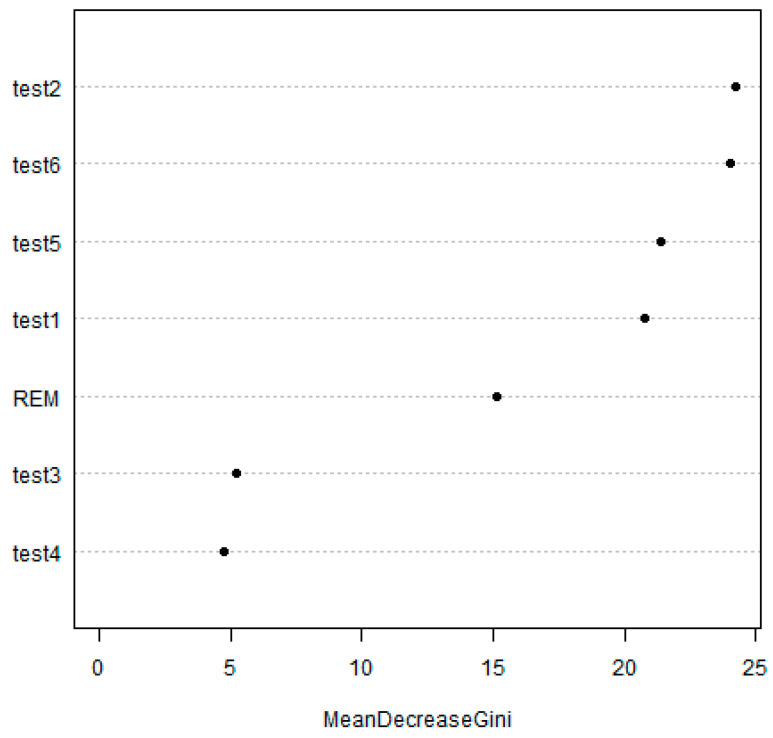
Variable importance in the Parkinson’s disease dementia (PDD) prediction model (random forest model). test1 = K-MMSE; test2 = K-MoCA; test3 = GDS; test4 = Global CDR score; test5 = sum of boxes in CDR; test6 = K-IADL; REM = Rapid eye movement and sleep behavior disorders.

The results of developing a PDD prediction model using random forest are presented in Figure 3. The random forest model estimates major neuropsychological indicators using the Gini impurity to differentiate AD from PDD. It was found that K-MoCA, K-IADL, the sum of boxes in CDR, K-MMSE, REM sleep behavior disorders, GDS, and Global CDR score, in the descending order of magnitude, were major neuropsychological indicators for estimating the diagnostic prediction capabilities in the random forest model. On the other hand, GDS was the least important indicator among all neuropsychological indicators for distinguishing between AD and PDD.

### 3.4. Performance of the PDD Prediction Model Based on Machine Learning

**Figure 4:**
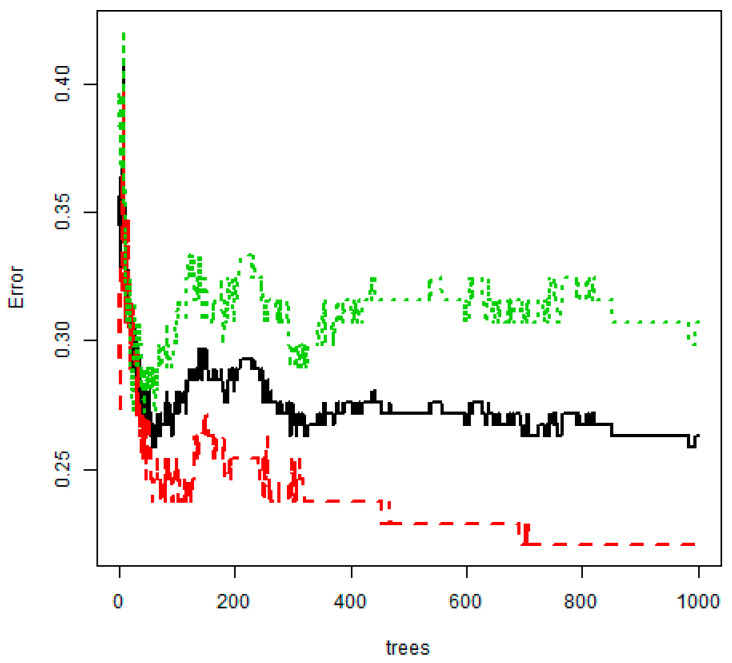
Out-of-bag error rate curve in random forest model (1000 trees). Black line = overall accuracy; red line = sensitivity; green line = specificity.

**Figure 5:**
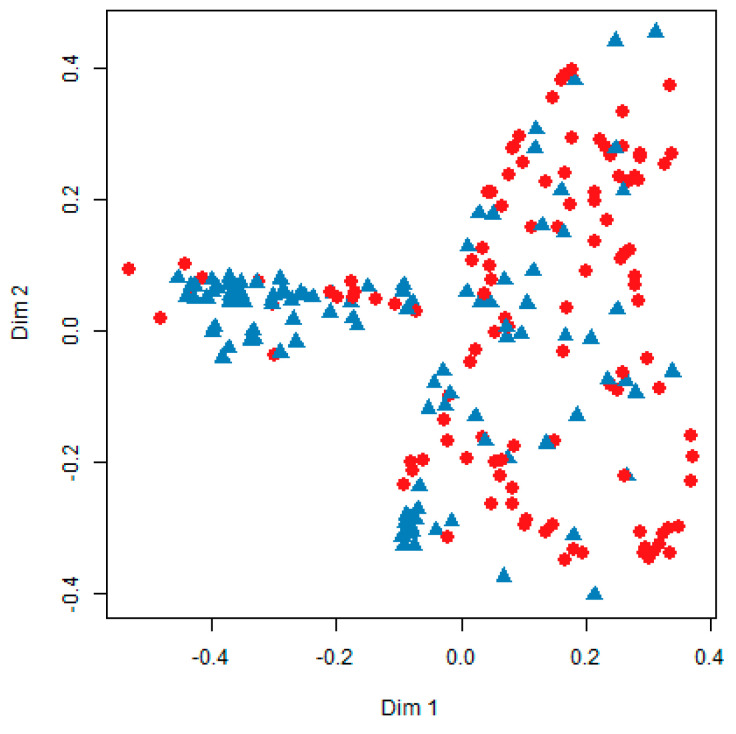
Multidimensional scale plots in random forest. Blue = AD; red = PDD.

The results of training data analysis showed that random forest using 1000 bootstrap samples had very high accuracy (75.1%) (Figure 4 and Figure 5). On the other hand, the accuracy of CART was 69.5% and that of the logistic regression model was 65.8%, the lowest. In the test data, random forest had the highest accuracy (73.3%), and the logistic regression model had the lowest accuracy (60.5%). Random forest had the highest accuracy in both training and test data. When ntree, the number of tree generation, and mtry were set as 1000 and 5, respectively, the final random forest model of this study had a sensitivity of 78.0% and a specificity of 70.0%.

## 4. Discussion

It is necessary to select a neuropsychological profile with high feasibility and predictive performance to easily distinguish PDD from other types of cognitive impairment in the health care field. To achieve this goal, studies exploring the diagnostic predictive capabilities of neuropsychological tests must be preceded. This study analyzed the diagnostic predictive capabilities of cognitive profiles that distinguished between AD and PDD using random forest. The results of this study show that K-MoCA, K-IADL, the sum of boxes in CDR, K-MMSE, REM sleep behavior disorders, GDS, and Global CDR score in the descending order of magnitude, were important diagnostic predictors. In particular, the K-MoCA was the most important neuropsychological index to distinguish AD from PDD.

An essential diagnostic element of PDD is the deterioration of cognitive functions after the onset of PD. These cognitive defects progress gradually and steadily in various domains, such as attention, executive functions, spatiotemporal abilities, memory, and language functions. Patients with PDD experience the defects in spatiotemporal abilities such as visual reasoning [39] in addition to the damage of executive functions [40], which implies the ability to establish concepts, discover rules, and solve problems from the early stage of the disease. It has been reported that patients with PDD have significantly less spatiotemporal executive functions than those with AD [41]. K-MMSE and K-MoCA are widely used in the health care field as screening tests to compare declined various cognitive functions by cognitive impairment type and confirm a comprehensive cognitive level. The results of this study show that K-MoCA had superior predictive power for distinguishing PDD and AD than K-MMSE. It is recommended to conduct K-MoCA first over other cognitive screening tests when conducting a neuropsychological test to distinguishing between PDD and AD in the future to increase the discriminative power. However, additional epidemiological studies should be conducted to verify that K-MoCA is the most important predictor for distinguishing between AD and PDD. It is because of a shortfall of machine learning that, although it has stronger prediction power than traditional regression models, such as logistic regression, it has a disadvantage that it does not allow to interpret the derived results. Therefore, future studies should employ hybrid-based machine learning, which has high predictive power and can interpret results.

The results of this study show that the prediction accuracy of random forest was higher than that of the logistic regression model and that of CART. The results of this study agree with the results of Byeon (2015) [30], who developed a model for predicting cognitive impairment in old age using random forest. Byeon (2015) highlighted that random forest had higher prediction accuracy than regression models or tree-based algorithms depending on one tree because random forest generates various decision trees from multiple bootstrap samples. Moreover, tree-based algorithms have the risk of overfitting because it may construct a node even if it is an outlier [42], whereas random forest maintains the tendency of trees with decreasing distribution to prevent overfitting [43]. Therefore, it is believed that using random forest will increase the prediction power while exploring major variables for predicting diseases than using regression models.

The importance of this study was that this study identified the priority of neuropsychological tests to distinguish between AD and PDD using reliable medical data conducted by the Korea Centers for Disease Control and Prevention. The limitations of the study were as follows. First, although the data source of this study was a national survey, the participants were sampled randomly. Secondly, the prediction model did not include a biomarker, a candidate marker, and genetic information. Since genes such as LRRK2 and G2385R are known to be the risk factors of PD and highly related to cognitive function, it will be possible to draw more clinically meaningful results when future studies develop prediction models while including genetic information, biomarkers, neuropsychological profile, Parkinson-related non-motor symptoms. Thirdly, input parameters did not include whether subjects took medication for PD, such as dopaminergics. Since medicine for PD affects not only behavioral symptoms but also the expression of cognitive symptoms, future prediction models shall consider or control the medication for PD. 

## 5. Conclusions

The results of this study show that Parkinson-related non-motor symptoms, such as REM sleep behavior disorders, and cognitive screening tests, such as K-MoCA, were highly accurate factors for predicting PDD. Based on these results, developing customized screening tests that can detect PDD in the early stage is required. Furthermore, it is believed that including biomarkers such as brain images or cerebrospinal fluid as input variables will be more useful for developing PDD prediction models in the future.

## Figures and Tables

**Table 1:** Characteristics of the participants based on type of dementia, chi-square test, *n* (%).

Factors	AD (*n* = 118)	PDD (*n* = 110)	*p*
Age			0.006
60–74 years old	46 (42.2)	63 (57.8)	
≥75 years old	72 (60.5)	47 (39.5)	
Gender			0.055
Male	33 (42.9)	44 (57.1)	
Female	85 (56.3)	66 (43.7)	
Education			0.638
Middle school graduate and below	88 (52.7)	79 (47.3)	
High school graduate and above	30 (49.2)	31 (50.8)	
Handless			0.547
Right hand	114 (51.6)	107 (48.4)	
Left hand	3 (60.0)	2 (40.0)	
Both hands	0	1 (100)	
Family PD history			0.034
No	74 (48.1)	80 (51.9)	
Yes	0	5 (100)	
Family dementia history			0.081
No	80 (51.6)	75 (48.4)	
Yes	8 (80.0)	2 (20.0)	
Pack year			0.441
1−20	2 (28.6)	5 (71.4)	
21−40	1 (25.0)	3 (75.0)	
41−60	1 (50.0)	1 (50.0)	
61 +	112 (52.6)	101 (47.4)	
Coffee-drinking			0.235
No	68 (54.8)	56 (45.2)	
Yes	1 (20.0)	4 (80.0)	
Carbon monoxide poisoning			0.623
No	84 (46.9)	95 (53.1)	
Yes	6 (54.5)	5 (45.5)	
Traumatic brain injury			0.381
No	85 (46.7)	97 (53.3)	
Yes	5 (62.5)	3 (37.5)	
Diabetes			0.921
No	85 (51.2)	81 (48.8)	
Yes	26 (52.0)	24 (48.0)	
Hypertension			0.660
No	58 (50.0)	58 (50.0)	
Yes	53 (53.0)	47 (47.0)	
Hyperlipidemia			0.902
No	102 (51.5)	96 (48.5)	
Yes	9 (50.0)	9 (50.0)	
Atrial fibrillation			0.522
No	106 (51.0)	102 (49.0)	
Yes	5 (62.5)	3 (37.5)	

**Table 2:** Characteristics of Parkinson’s non-motor symptoms and neuropsychological tests by dementia type, independent *T*-test, *n* (%).

Characteristics	AD (*n* = 118)	PDD (*n* = 110)	*P*
REM sleep behavior disorders			0.003
No	74 (67.3)	36 (32.7)	
Yes	17 (23.9)	54 (76.1)	
Depression (GDS)			0.004
No	53 (62.4)	32 (37.6)	
Yes	22 (37.9)	36 (62.1)	
K-MMSE, mean ± SD	17.6 ± 5.9	18.5 ± 5.6	0.283
K-MoCA, mean ± SD	9.6 ± 5.5	11.7 ± 5.4	0.033
Global CDR score, mean ± SD	1.2 ± 0.8	1.0 ± 0.7	0.011
Sum of boxes in CDR, mean ± SD	7.2 ± 6.6	5.2 ± 4.9	0.037
K-IADL, mean ± SD	3.0 ± 5.1	3.1 ± 5.5	0.963

REM = rapid eye movement; GDS = the Geriatric Depression Scale; K-MMSE = the Korean version of Mini Mental State Examination; K-MoCA = Korean version of Montreal Cognitive Assessment; CDR = Clinical Dementia Rating; K-IADL = Korean version of Instrumental Activities of Daily Living.
